# Measles cluster at a university in the United Kingdom

**DOI:** 10.1186/1756-0500-7-744

**Published:** 2014-10-22

**Authors:** Jenny Gamblin, Jane Maund, Iain Blair, Stuart C Clarke

**Affiliations:** Public Health England, Hampshire and Isle of Wight Health Protection Unit, Fareham, Hampshire, UK; Faculty of Medicine, University of Southampton, Southampton, UK

**Keywords:** Measles, Disease outbreaks, Universities, Immunization

## Abstract

**Background:**

Measles remains an infection of public health importance. We describe a cluster of measles in a university setting between April and May 2007.

**Case presentation:**

The outbreak took place over a period of six weeks and involved nine students, eight of whom lived in halls of residence. Due to the potential for significant spread in an institutional setting, a public health investigation was initiated to identify the source of the outbreak. Follow up of cases was undertaken proactively with the university and local general practitioners. Salivary fluid test kits and questionnaires were sent to suspected cases. Seven salivary test kits were returned, but only one questionnaire was returned. Four cases were confirmed as measles. Although seven students had been previously immunised, immunity was only demonstrated in three.

**Conclusions:**

Public health investigations were hampered by the poor return rate of questionnaire and oral fluid sampling kits despite proactive follow up by public health staff, as well as the break for summer vacation. Nevertheless, our report highlights the public health effort in investigating such outbreaks and the importance of reinforcing immunisation to college and university students.

## Background

Measles remains an infection of public health importance [1]. It is a highly infectious viral illness transmitted via droplet infection. Almost all who are infected develop symptoms which include fever and a distinctive rash. The incubation time is between 10 and 14 days although an infected person is contagious from four days before until four days after the rash appears. Most people recover within 7–10 days, but complications can include ear infections, pneumonia and diarrhoea. Measles has become rare because of MMR (mumps, measles and rubella) immunisation but there have been recent outbreaks in children who have not been immunised. Measles outbreaks in the university setting are reported rarely [2–6]. Here we report an outbreak of measles in an English university.

## Case presentation

The outbreak took place in an English university over a period of six weeks (15^th^ April to 25^th^ May 2007) involving nine students in their first academic year, eight of whom lived in university halls of residence (Table 1). Local Health Protection Agency (HPA) staff (now part of Public Health England) were alerted to the possibility of a cluster of measles on 3^rd^ May 2007 and all cases were investigated according to standard case definitions. Oral fluid sampling kits for the detection of IgM/IgG were mailed to cases with a covering letter and stamped addressed envelope for direct return to the HPA national reference laboratory. These were requested to be taken as soon as possible so that suspected cases that fitted the case definitions could be laboratory confirmed [7]. If samples were suggestive of recent measles, then genotyping was performed. Proactive follow up of cases took place with the aim of gaining return of salivary kits and questionnaires.

**Table 1:** **Epidemiological findings and laboratory results**

Case	Date of onset	Epidemiological link	Immunisation status	Laboratory result		Typing	Measles status
				IgM	IgG		
1	15^th^ April 2007	Social contact with case 4. Lived in halls of residence A. Recent travel elsewhere within the South East of England.	Not immunised	Positive	Not known	Not available	Confirmed measles
2	30^th^ April 2007	Student but lived outside university setting.	MMR × 2	Negative	Positive	N/A	Previous immunity
3	30^th^ April 2007	Lived in halls of residence A. Recent travel to London.	Not immunised	Positive	Negative	D4	Confirmed measles
4	3^rd^ May 2007	Social contact with case 1. Lived in halls of residence A.	MMR × 1	Positive	Positive	Not available	Confirmed measles
5	10^th^ May 2007	Lived in same flat as cases 6 and 7 within halls of residence B.	MMR × 1	Negative	Positive	N/A	Previous immunity
6	12^th^ May 2007	Lived in same flat as cases 5 and 7 within halls of residence B.	MMR × 1, MR × 1	-	-	N/A	Unconfirmed
7	13^th^ May 2007	Lived in same flat as cases 5 and 6 within halls of residence B.	MMR × 2	-	-	N/A	Unconfirmed
8	15^th^ may 2007	Lived in halls of residence A.	MMR × 1, MR × 1	Positive	Positive	D4	Confirmed measles
9	25^th^ May 2007	Lived within halls of residence B.	MMR x 2	Negative	Positive	N/A	Previous immunity

Contacts were defined as those who had had room contact with a confirmed, or epidemiologically linked, or suspected index case from four days before to five days after the appearance of the rash in the case. Risk assessment of contacts was based on the type of contact, measles immunity, immunosuppression, age, and pregnancy. No cases or contacts fell into any risk groups.

Information regarding the symptoms of measles disease, protection of contacts and promotion of the MMR vaccination was disseminated to those students living within the same halls of residence or attending the same lecture group. This was also sent to NHS Direct (part of the National Health Service), three local general practitioner (GP) practices, including the University Health Centre, and to Out of Hours GP services. However, as more cases were identified, the information was further distributed to all members of the University via the intranet and an outbreak meeting was convened. After the first two cases, a detailed questionnaire was designed and sent to all cases. Although this was sent through official university channels only one questionnaire was returned.

Case 1 was a confirmed measles case but was identified at a later stage of the outbreak and had been in social contact with case 4 (confirmed). Case 1 had no known contact with measles but had travelled to another town within the South East of England a week before onset of illness. No cases of measles had been recently reported within that town. The case had not been immunised. This case remained within the university setting (halls of residence A) for five days after onset of illness. Salivary antibody testing for case 1 confirmed recent measles disease.

Case 2 resulted in declaration of a probable outbreak. The case presented with symptoms of fever, rash and conjunctivitis but was subsequently determined to have received two doses of MMR vaccine. This case lived outside the university setting and had attended lectures as normal during the illness, prior to the case being notified. The case had not had known contact with measles and no epidemiological link with the other cases could be identified. On salivary testing the case was found to be already immune to measles.

Case 3 was a confirmed measles case and was the first notified case. This case lived in halls of residence A and had travelled to London whilst ill. The case had not been immunised and salivary antibody testing confirmed recent measles disease, genotype D4.

Case 4 was a confirmed measles case and was the only case to be hospitalised with symptoms of general malaise, rash and conjunctivitis. The patient had an uneventful recovery. The case also lived in halls of residence A. The case had received one MMR vaccination but recent measles disease was confirmed on salivary testing. It was ascertained that case 4 had been in social contact with case 1 (also confirmed).

Case 5 was identified through case 6, lived within the same flat as cases 6 and 7 (in halls of residence B), and had had symptoms of measles two days prior to case 6. Case 5 had been immunised once with MMR vaccine and salivary testing revealed previous immunity to measles.

Case 6 was notified with symptoms of rash and fever and had received one dose of MR vaccine and one dose of MMR vaccine. A salivary testing kit was sent to the case but was not returned. Case 6 lived in the same flat as cases 5 and 7 within halls of residence B.

Case 7 lived in the same flat as cases 5 and 6 within halls of residence B and developed symptoms of headache, sore eyes, cold symptoms and fever. An epidemiological link for case 7 could not be identified since the incubation period from contact with cases 5 and 6 was too short. This case had received two doses of MMR. A salivary testing kit was sent to the case but was not returned.

Case 8 was a confirmed measles case and was notified with symptoms of rash and fever and had received one dose of MR vaccine and one dose of MMR vaccine. On salivary testing, recent measles was confirmed and typed as genotype 4. The case lived at the same residential complex as cases 1, 3 and 4 (all confirmed cases).

Case 9 was notified with symptoms of rash, fever and sore eyes. The case lived at the same residential complex as cases 5, 6 and 7 and shared the same lecture group as case 7. This case had been immunised twice with MMR vaccine. On salivary testing the case was found to have previous measles immunity.

## Conclusion

In summary, four cases of recent measles were confirmed by the laboratory but genotyping information was only available for two cases, and both were identified as genotype D4. Two of the confirmed cases had not been immunised, one had received 1 MMR vaccination and one had received one MMR and one MR. Unfortunately, two cases did not return salivary testing kits and the remaining three cases were identified as having previous immunity to measles. Measles disease was only confirmed in hall of residence A. Cases associated with hall of residence B had either previous immunity or salivary testing kits were not returned. The apparent symptoms of measles disease associated with hall of residence B may have reflected increased awareness of the signs and symptoms of this disease.

The efficacy of a single dose of measles-containing vaccine is around 90% and a second dose of a measles-containing vaccine should protect most of those who do not respond to the first dose [8]. Interestingly, case 8 had received one MR vaccination and one MMR vaccination but laboratory testing confirmed recent measles disease.

Follow-up of cases in an outbreak scenario is of most importance. A proactive role is required in order to gain complete information on clinical symptoms, case contacts and immunisation history. Despite proactive follow up of cases during this cluster, including close liaison with University colleagues and general practitioners, the epidemiological investigation was hampered by the non-return of salivary kits from two possible cases and the return of only one questionnaire from the nine possible cases. Although it was our aim to gain much of this information through the detailed questionnaire, the poor return rate meant that this information remained incomplete. In retrospect, the questionnaire could have been distributed as soon as the cases were notified, particularly as the end of term was fast approaching. Only one questionnaire out of a total of nine was returned which severely hampered epidemiological investigation. Follow-up is also required in order to gain a laboratory confirmation.

The case cluster of measles reported herein highlights the significance of reinforcing immunisation to new college and university students. The importance of MMR vaccination is emphasised in university welcome guides whereby students are advised to seek immunisation prior to commencement of the academic term. For those who have not done so, information with respect to obtaining MMR is also provided.

## Consent

This work was carried out as part of routine public health investigations by the Health Protection Agency (now part of Public Health England) and therefore written informed consent was not required. Public Health England has approval under the Health and Social Care Act 2001 to process confidential patient information for public health purposes (see http://www.legislation.hmso.gov.uk/si/si2002/20021438.htm).
